# What Is the Role of *Archaea* in Plants? New Insights from the Vegetation of Alpine Bogs

**DOI:** 10.1128/mSphere.00122-18

**Published:** 2018-05-09

**Authors:** Julian Taffner, Armin Erlacher, Anastasia Bragina, Christian Berg, Christine Moissl-Eichinger, Gabriele Berg

**Affiliations:** a Institute of Environmental Biotechnology, Graz University of Technology, Graz, Austria; b Institute of Plant Sciences, University of Graz, Graz, Austria; c Department of Internal Medicine, Medical University of Graz, Graz, Austria; National Institute of Advanced Industrial Science and Technology

**Keywords:** *Archaea*, plant microbiome, plant-microbe interactions

## Abstract

The *Archaea* represent a significant component of the plant microbiome, whereas their function is still unclear. Different plant species representing the natural vegetation of alpine bogs harbor a substantial archaeal community originating from five phyla, 60 genera, and 334 operational taxonomic units (OTUs). We identified a core archaeome for all bog plants and ecosystem-specific, so far unclassified *Archaea*. In the metagenomic data set, *Archaea* were found to have the potential to interact with plants by (i) possible plant growth promotion through auxin biosynthesis, (ii) nutrient supply, and (iii) protection against abiotic (especially oxidative and osmotic) stress. The unexpectedly high degree of plant specificity supports plant-archaeon interactions. Moreover, functional signatures of *Archaea* reveal genetic capacity for the interplay with fungi and an important role in the carbon and nitrogen cycle: e.g., CO_2_ and N_2_ fixation. These facts reveal an important, yet unobserved role of the *Archaea* for plants as well as for the bog ecosystem.

**IMPORTANCE** *Archaea* are still an underdetected and little-studied part of the plant microbiome. We provide first and novel insights into *Archaea* as a functional component of the plant microbiome obtained by metagenomic analyses. *Archaea* were found to have the potential to interact with plants by (i) plant growth promotion through auxin biosynthesis, (ii) nutrient supply, and (iii) protection against abiotic stress.

## Abstract (precis)

*Archaea* are still an underdetected and little-studied part of the plant microbiome. We provide first and novel insights into *Archaea* as a functional component of the plant microbiome obtained by metagenomic analyses. *Archaea* were found to have the potential to interact with plants by (i) plant growth promotion through auxin biosynthesis, (ii) nutrient supply, and (iii) protection against abiotic stress.

## INTRODUCTION

During the last several decades, our picture of the diversity and metabolic potential of the *Archaea* in a wide variety of environments has been revolutionized (1, 2). For example, *Archaea* represent an important component of the human and plant microbiome, where their impact on their host is still unclear (3, 4). Within plants, *Archaea* are differently distributed (4). They have often been found in the rhizosphere and endosphere but rarely in the phyllosphere, which can be explained by the different abiotic conditions in these microenvironments (5–10). Besides abiotic factors and adaptation to chronic energy stress, archaeal colonization depends on biotic factors such as competition with bacteria, which might have led to microniche differentiation (11). Even though factors influencing archaeal functionality under specific anaerobic conditions in rice roots have been analyzed (9, 12), their ecological roles and interactions with plants remained largely unclear. The fact that most of *Archaea* are difficult to cultivate and that plant-associated archaeal pathogens are currently not known may be attributed to the lack of knowledge. However, due to their ubiquitous occurrence on healthy plants, we assume that *Archaea* interact positively with plants.

Plants harbor highly diverse and to a certain extent species-specific microbiomes (13–15). These microbiomes play an essential role for the plant as they can alter plant growth, productivity, adaptation, diversification, and health (16, 17). Especially in bog ecosystems, we have shown that plants and microbiota are closely interlinked (18–21). Bogs are one of the oldest terrestrial ecosystems on Earth (22); their functioning under extreme conditions is a result of a long period of coevolution between plants and microorganisms. Bog ecosystems fulfill important functions for the whole biosphere, as a reservoir for freshwater and for soil organic matter, acting as a carbon sink (23, 24). Since most of these ecosystems are extremely poor in accessible nutrients because they rely on rain water only (ombrotrophic lifestyle), plant-associated bacteria are known to play a crucial role in nutrient supply and cycling (25). Furthermore, raised bogs show a unique biodiversity, harboring a unique and highly specialized flora and fauna. The vegetation is often dominated by *Sphagnum* mosses, which play an important role in global carbon cycling and even in global climate (26). Especially the bacterial community associated with *Sphagnum* shows a supportive effect on plant health (18, 19), productivity (25), and peatland nutrient cycling (23, 27). The *Sphagnum* bacterial community is, to an extraordinary degree, host specific, is vertically transmitted, and contains different functional patterns that strongly support bog functioning under extreme environmental conditions: e.g., pH (highly acidic), nutrient availability (extremely low), and high water saturation (20, 28). In addition to *Sphagnum* mosses, there is diverse and well-adapted vegetation shaping this ecosystem: e.g., acidophytic bryophytes (Polytrichum strictum and Aulacomnium palustre), graminoids (Eriophorum vaginatum and Carex nigra), dwarf shrubs (Andromeda polifolia and Vaccinium oxycoccus), small trees (Pinus mugo), and lichens (e.g., Cladonia fimbriata). All components of the vegetation are embedded into *Sphagnum* mosses, forming the oxic acrotelm layer, which consists mostly of living plant material (see Fig. S1 in the supplemental material), in contrast to the anoxic catotelm, which consists of dead plant material (peat). The understanding of plant-microbe interactions in this specific bog environment still misses a relevant jigsaw puzzle piece: *Archaea* and their role in supporting functioning of this extreme ecosystem yet remain mostly unexplored. *Archaea* are expected to play an important role in nutrient supply (29) and stress protection (30).

10.1128/mSphere.00122-18.1 FIG S1  Potential archaeal colonization pattern associated with vegetation of alpine raised bog-systems, based on our findings. Most representative vegetation forms are graminoids, dwarf shrubs, and mosses/sphagna (shown from left to right, respectively). Archaeal colonization is indicated as a red-shaded gradient. The acrotelm is the upper layer of a peat bog, affected by aerobic decomposition. The catotelm is the lower anaerobic layer of a peat bog, in which organic material stays undecomposed. Download FIG S1, TIF file, 2.9 MB. Copyright © 2018 Taffner et al. 2018 Taffner et al. This content is distributed under the terms of the Creative Commons Attribution 4.0 International license.

The objective of our study was to find out if plants harbor specific archaeal communities and to identify potential modes of interaction of *Archaea* on plants in general. Another more specific objective was to integrate *Archaea* into the concept of the microbiome-driven functioning of the bog ecosystem. Therefore, we studied the archaeome of 46 plant samples originating from the green and oxic acrotelm layer, which represent the typical bog vegetation of alpine bogs. Samples were taken in Rotmoos and Pürgschachen Moor (Austria) and analyzed by a complementary approach of metagenomics and specific sequencing of the V4 region of the 16S rRNA gene fragment.

## RESULTS

### Composition of *Archaea* associated with bog vegetation.

**FIG 1:**
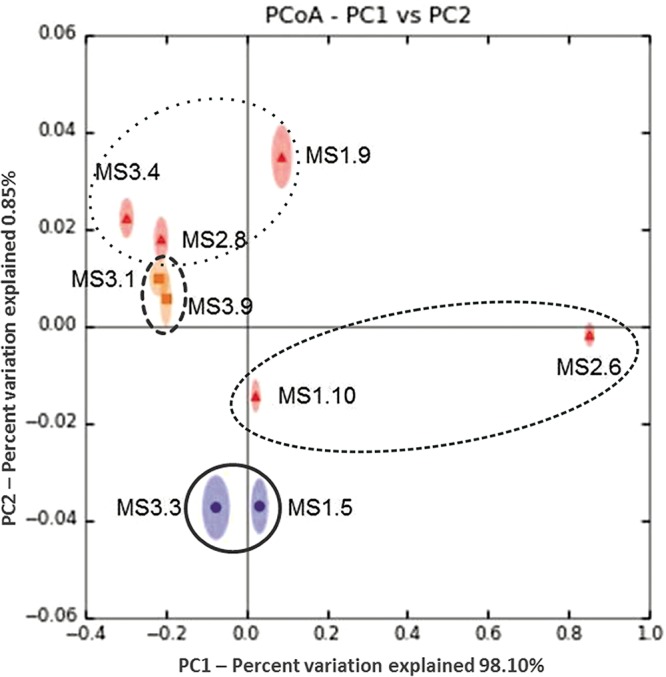
Comparison of archaeal communities associated with bog vegetation by principal-coordinate analysis (PCoA). The PCoA plot is based on a Bray-Curtis distance matrix of the 16S rRNA gene amplicon libraries and supported by 100 jackknife data resamplings using 1,000 sequences per library. The ellipses with symbols in the center show single samples with their IDs. Sample descriptions are as follows: Vaccinium oxycoccus (MS1.5), S. magellanicum (MS1.9, MS2.8), Polytrichum strictum (MS1.10), P. commune (MS2.6), Eriophorum vaginatum (MS3.1), V. myrtilus (MS3.3), S. capillifolium (MS3.4), and Molinia caerulea (MS3.9). Samples belonging to the clade Eudicotyledons are shown as solid circles circled by a solid line, samples of the clade Monocotyledons are shown as squares circled by a dashed line, and the samples of the clade *Bryopsida* (separated into the classes *Sphagnopsida* and *Polytrichopsida*) are shown as triangles circled by dotted and smaller dotted lines, respectively, in the center of the ellipses. Variation explained by each principal coordinate (PC) is defined on the plot.

16S rRNA amplicon analysis of 46 samples, including bryophytes, vascular plants, and lichens, resulted in amplicons for 41 samples (Table 1). Out of an overall data set of 305,430 sequences, 23,400 sequences (7.7%) were annotated to *Archaea* and clustered into 334 operational taxonomic units (OTUs). The data set was further normalized to 1,000 sequences per sample, resulting in the exclusion of 31 samples containing fewer. The estimated sequencing coverage for *Archaea* varied from 44.2% to 100%, with a mean value of 83.2%. The relative archaeal abundance differed from plant species to species and ranged from 0% to 33%. The highest relative abundances were detected in the samples of deep-rooted plants like blueberry (Vaccinium myrthillus [33%]) and cranberry (V. oxycoccus [31.7%]) and monocots like tussock cottongrass (Eriophorum vaginatum [29.1%]) and purple moor-grass (Molinia caerulea [20.2%]). However, also moss species harbored a substantial proportion of archaeal signatures: Polytrichum commune (25.4%), Sphagnum capillifolium (24.6%), S. magellanicum (18.2%), and P. strictum (16.7%). In a principal-coordinate analysis (PCoA) performed with representative species, archaeal communities in samples belonging to the class-level eudicots and monocots formed a distinct cluster, whereas samples belonging to the classes of *Sphagnopsida* and *Polytrichopsida* were more widespread (Fig. 1). Overall, the eudicot samples were more separate from the other groups, showing a lower diversity.

**TABLE 1:**
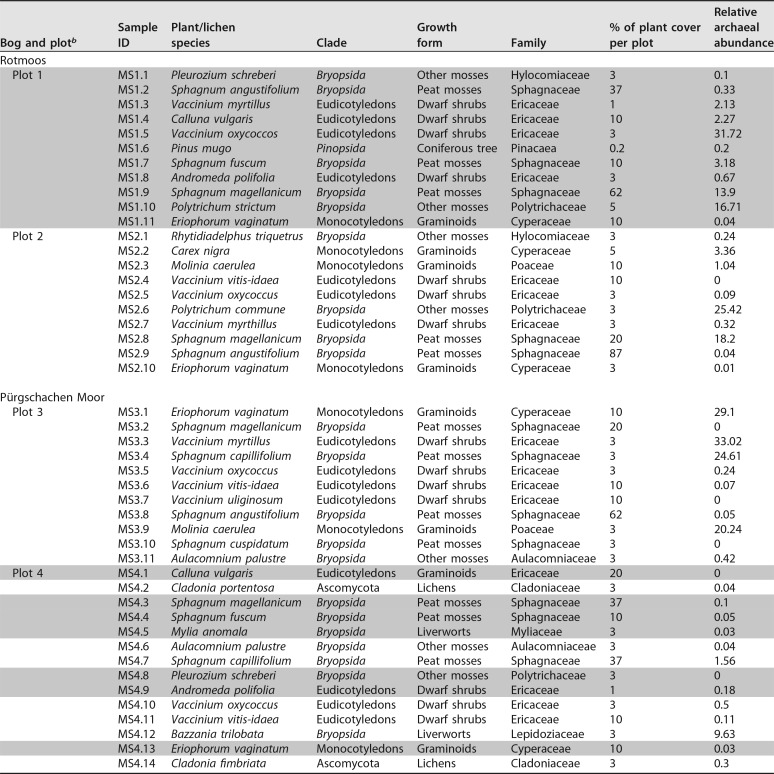
List of the complete set of 46 samples of overall representative vegetation of the bog, regarding their sampling location and plant coverage per plot^a^

a All samples were analyzed with 16S rRNA sequencing, resulting in the displayed relative abundance of *Archaea* of the prokaryotic microbiota. Gray-shaded samples were additionally used for metagenomic studies, whereas the samples MS1.1 and -4.8, MS1.7 and -4.4, MS1.9 and -4.3, MS1.11 and -4.13, MS1.4 and -4.1, and MS1.8 and -4.9 were pooled prior to sequencing.

b Plot locations are as follows: plot 1, N47 41.029 E15 09.284, 695 m; plot 2, N47 41.059 E15 09.269, 695 m; plot 3, N47 34.835 E14 20.390, 632 m; and plot 4, N47 34.815 E14 20.482, 632 m.

**FIG 2:**
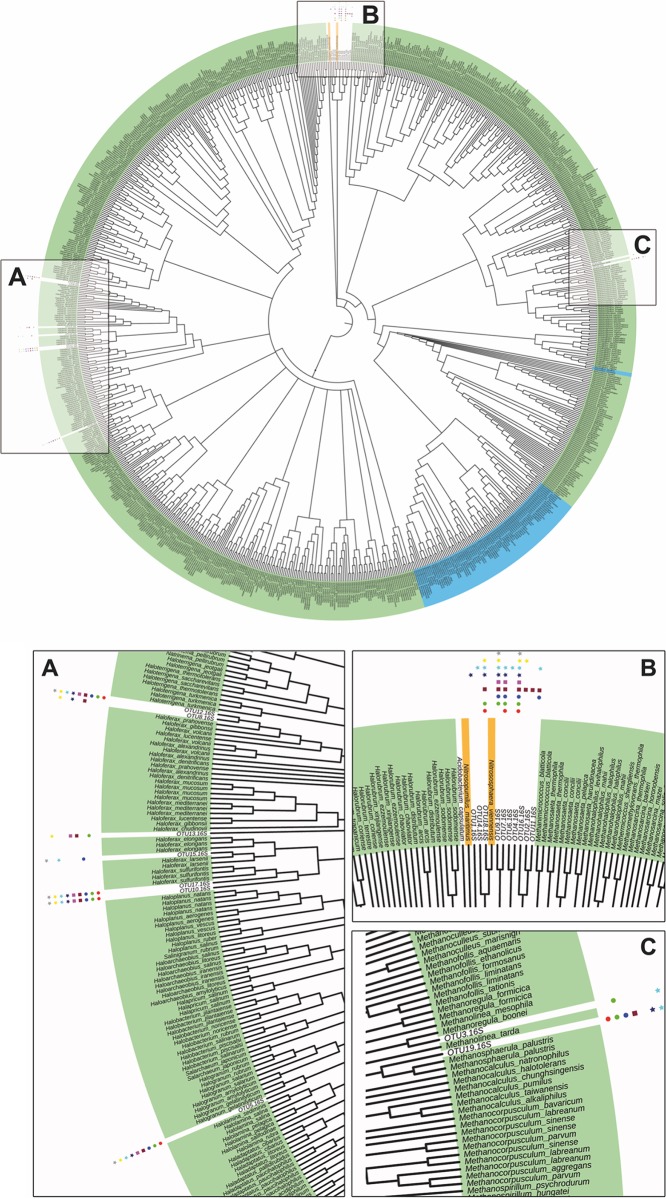
16S rRNA gene sequence-based DNA distance maximum likelihood tree of archaeal OTUs, of Acidobacterium capsulatum strain ATCC 51196 (NR_074106.1, outgroup), and the complete archaeal RefSeq database. Based on the neighboring clades, OTUs were taxonomically identified. Three main clusters (A to C) were formed, where the highest occurrence was found in the underrepresented phylum in the RefSeq database of *Euryarchaeota*. Symbols represent occurrence of the OTU, as indicated in the legend to Fig. 3. Green, *Euryarchaeota*; blue, *Crenarchaeota*; orange, *Thaumarchaeota*.

**FIG 3:**
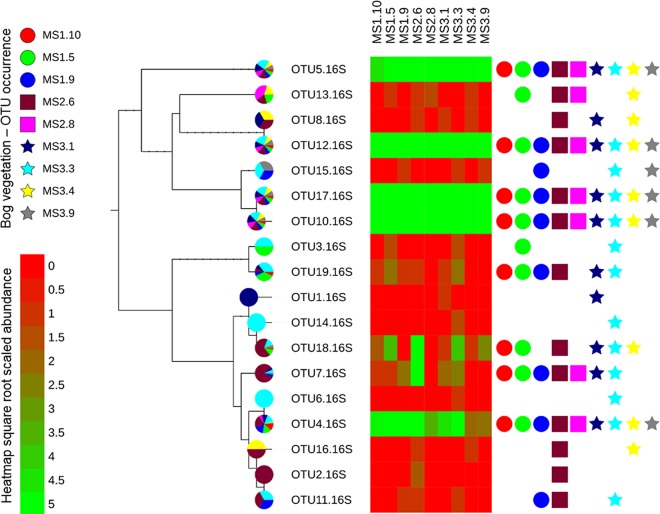
Pruned DNA distance maximum likelihood phylogenetic tree of archaeal 16S rRNA OTUs associated with bog vegetation: Vaccinium oxycoccus (MS1.5), *Sphagnum magellanicum* (MS1.9 and MS2.8), Polytrichum strictum (MS1.10), P. commune (MS2.6), Eriophorum vaginatum (MS3.1), V. myrtilus (MS3.3), S. capillifolium (MS3.4), and Molinia caerulea (MS3.9). Phylogenetic relationships are shown for 16S rRNA sequences representing the structure of archaeal OTUs. Pie charts show the OTU proportional distribution between the samples. Symbol charts represent the occurrence. Heat map abundance is based on square root scaled abundance: in order to discriminate between the lower-abundance groups, the upper heat map cap was set to 5.

In total, based on the 16S rRNA gene data set, the archaeome associated with the bog vegetation showed low taxonomic diversity. A maximum likelihood phylogenetic tree was constructed based on the archaeal 16S rRNA amplicon sequences aligned to the complete archaeal 16S rRNA gene RefSeq database (Fig. 2). Based on the DNA distance maximum likelihood algorithm using 1,000 bootstraps, three main phylogeny clusters (A to C) were formed. Phylogenetic neighbor comparison allowed taxonomical identification, showing the highest abundant OTUs belonging to the phylum of *Euryarchaeota* could be assigned to the genera *Haloferax* (OTUs 8, 10, 12, 13, 15, and 17) and *Halogranum* (OTU 5), forming cluster A. The most abundant representative species were phylogenetically related to Haloferax sulfurifontis, Haloferax prahovense, and Halogranum gelatinilyticum. The second cluster B was formed within the phylum *Thaumarchaeota*, whereas OTUs were closely related to Nitrososphaera viennensis (OTUs 14 and 18) and Nitrosopumilus maritimus (OTU 1). Within cluster B, some OTUs (2, 4, 6, 7, 9, 11, and 16) formed a distinct branch, which was phylogenetically more distantly related to the classified *Thaumarchaeota* species of the RefSeq database. Furthermore, a third cluster, C, could be assigned to the *Euryarchaeota* species Methanoregula boonei (OTU 3) and Methanosphaerula palustris (OTU 19). A direct comparison of all OTUs revealed that the most abundant OTUs (4, 5, 10, 12, and 17) were present in all samples, forming an archaeal core microbiome (Fig. 3). Samples of P. commune (MS2.6) and V. myrtillus (MS3.3) showed the highest diversity of OTUs, whereas S. magellanicum (MS2.8) and M. caerulea (MS3.9) showed the lowest diversity.

**FIG 4:**
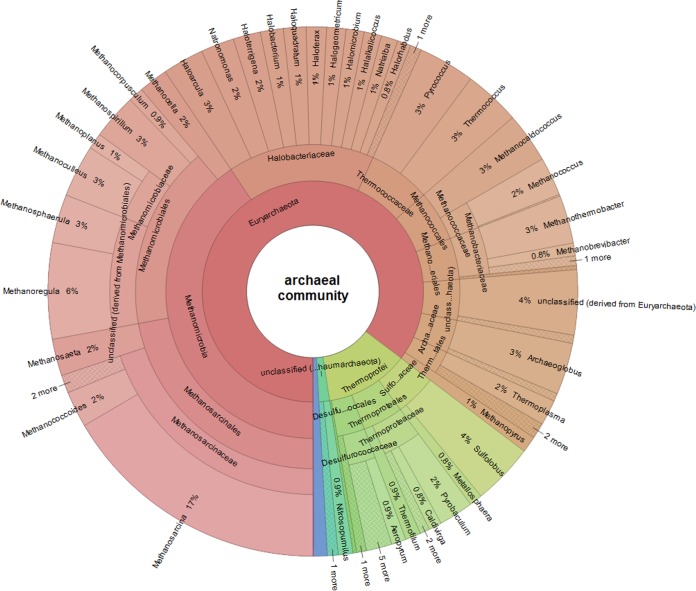
Krona chart representing taxonomic composition of the whole archaeal community associated with bog vegetation, revealed by metagenome sequencing. Abundances of archaeal genera are displayed relative to all sequences assigned to *Archaea* of the whole data set of 12 metagenomes (736,325 sequences). Metagenomes were obtained from 12 different plant species: Pleurozium schreberi, Sphagnum angustifolium, Vaccinium myrtilus, Calluna vulgaris, V. oxycoccus, Pinus mugo, S. fuscum, Andromeda polifolia, S. magellanicum, Polytrichum strictum, Eriophorum vaginatum, and Molinia anomala.

The analysis of the archaeal sequences based on the 12 metagenomes revealed as expected a more detailed phylogenetic structure than the 16S rRNA amplicon data set (Fig. 4). Overall, the archaeal community made up 0.2% to 0.7% (842,752 hits) of all prokaryotic abundance (189,394,645 hits). The archaeal phylum *Euryarchaeota* was the dominant group accounting for 85.4% of the whole archaeal community, followed by *Crenarchaeota* (12.3%) and *Thaumarchaeota* (1.6%). *Archaea* belonging to the phylum *Korarchaeota* (0.8%) and *Nanoarchaeota* (0.1%) were less represented. At the class level, the main annotated groups belonged to *Methanomicrobia* (40.6%), Halobacteria (16.2%), and Thermoprotei (12.3%). In total, 60 different archaeal genera could be determined. The most abundant genera could be identified as *Methanosarcina* (16.6%), *Methanoregula* (5.9%), *Sulfolobus* (3.8%), *Pyrococcus* (3. 5%), and *Thermococcus* (3.4%). In contrast to the 16S rRNA gene data set, *Archaea* of the genus *Haloferax* were less represented (1.2%).

### Metagenome-inferred function of *Archaea* associated with bog vegetation.

Functional analysis of 12 normalized metagenomes of the bog vegetation resulted in 285,058 archaeal hits, which could be assigned to certain functional subsystems of SEED database. Out of these annotations, a significant number of hits represented primary metabolic functions of *Archaea* (carbohydrates, 21. 6%; central carbohydrate metabolism, 7.6%; amino acids and derivatives, 20.1%; fatty acids, lipids, and isoprenoids, 2.3%; and cofactors, vitamins, prosthetic groups, and pigments, 6.4%). Besides functions of the central carbohydrate metabolism, 1.6% and 2.2% of archaeal functional hits were assigned to the fermentation and one-carbon metabolism subsystem, respectively. Functional signatures of *Archaea* involved in nutrient cycling were found as well, like signatures for CO_2_ fixation, which were highly abundant (0.7%). In contrast, the functions assigned to subsystems of nitrogen fixation were detected with less than 0.1%. On the top SEED level, 1% were detected as contributing to nitrogen metabolism. Thereby the most abundant subsystem was found to contribute to ammonia assimilation (0.7%). Interestingly, the number of genetic attributes encoding a stress response was high (2%). Especially the abundance of subsystems involved in oxidative stress response was at 0.9%. Further subsystems contributing to the archaeome’s stability, like attributes involved in DNA repair (2.3%) and osmotic stress (0.3%), were also abundant. In addition, archaeal subsystems involved in motility and chemotaxis (1.4%), like functions assigned to flagellar motility (0.2%), functional signatures for glycogen degradation, which is mainly found in fungi (0.3%), and interestingly subsystems involved in the plant-hormone biosynthesis of auxin (0.7%) were also found (see Tables S2 and S3 in the supplemental material). Nucleotide sequences for genes involved in auxin biosynthesis (EC 2.4.2.18, EC 5.3.1.24, EC 4.2.1.20, and EC 1.4.3.4) were further analyzed by using blastx. The taxonomic distribution of these genes among *Archaea* revealed a domain-wide distribution for EC 2.4.2.18, EC 5.3.1.24, and EC 4.2.1.20 (830, 213, and 877 archaeal hits, respectively), whereas the genes for monoamine oxidase (EC 1.4.3.4) were less represented and mainly found in *Euryarchaeota* (56 archaeal hits).

To further study the plant specificity of *Archaea* and archaeal functions on bog vegetation, the functional distribution among the clades of monocotyledons and eudicotyledons and the class of *Bryopsida* was analyzed. In total, at the top SEED level, monocotyledons (Eriophorum vaginatum) and eudicotyledons (Vaccinium myrtillus, Calluna vulgaris, V. oxycoccus, and Andromeda polifolia) showed a similar distribution of abundance of archaeal functions (32% and 29%, respectively). Whereas 39% of all assigned archaeal functions belonged to the *Bryopsida* samples (Polytrichum strictum, Pleurozium schreberi, Sphagnum angustifolium, S. fuscum, S. magellanicum, and Mylia anomala). For the *Bryopsida*, a distinct predominance of attributes involved in subsystems of the regulation and cell signaling, the cell division and cell cycle, phosphorus metabolism, DNA metabolism, and the nucleosides and nucleotides were detected. On SEED level 2, *Bryopsida* showed an increased abundance of archaeal functional groups responsible for the response to osmotic stress and purine metabolism, compared to the two other groups. However, functions of the oxidative stress response, nitrogen metabolism, and especially nitrate and nitrite ammonification were mostly found in monocotyledons. Although eudicotyledons constantly showed a reduced relative abundance of archaeal functions, subsystems assigned to allantoin utilization in nitrogen metabolism were exclusively detected in eudicots. In more detail, on the plant species level, archaeal functions associated with auxin biosynthesis, response to oxidative stress, CO_2_ fixation, and DNA repair were most abundant in Sphagnum fuscum, S. magellanicum, Pleurozium schreberi, Polytrichum strictum, Mylia anomala, and Eriophorum vaginatum. In general, in samples of Vaccinium myrtillus and Pinus mugo, a low abundance of these archaeal signatures was found. Functional signatures involved in glycogen degradation were especially represented in Sphagnum angustifolium, Polytrichum strictum, and Sphagnum fuscum.

**FIG 5:**
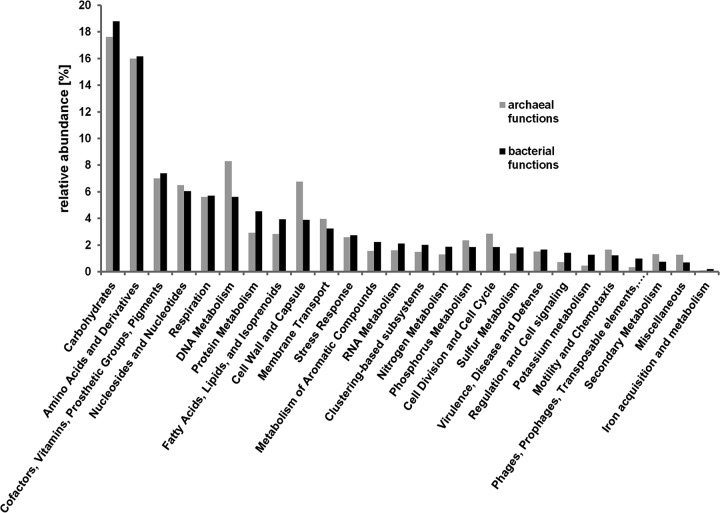
Comparison of functional compositions of archaeal (gray) and bacterial (black) communities associated with bog vegetation: Pleurozium schreberi, Sphagnum angustifolium, Vaccinium myrtilus, Calluna vulgaris, V. oxycoccus, Pinus mugo, S. fuscum, Andromeda polifolia, S. magellanicum, Polytrichum strictum, Eriophorum vaginatum, and Molinia anomala. Functional signatures were obtained from metagenomes, annotated using functional subsystems of SEED database, and processed with MG-RAST. Bar charts represent relative abundance of archaeal and bacterial functions of all functions annotated to *Archaea* (285,058 hits) and *Bacteria* (14,157,480 hits), respectively.

The functional composition of *Archaea* in the bog ecosystem was further compared with the composition of bacterial functions (Fig. 5). The distributions of functions within the domains *Archaea* and *Bacteria* were similar, with the most dominant subsystems representing carbohydrate and amino acid metabolism, as the most important biochemical processes, although there were some functions that were represented relatively higher in *Archaea* than in *Bacteria*—like subsystems corresponding to DNA metabolism (DNA repair) and cell wall and capsule. Functional groups belonging to the less dominant subsystems cell division and cell cycle, motility and chemotaxis, and secondary metabolism (auxin biosynthesis) were also relatively more represented by *Archaea*.

**FIG 6:**
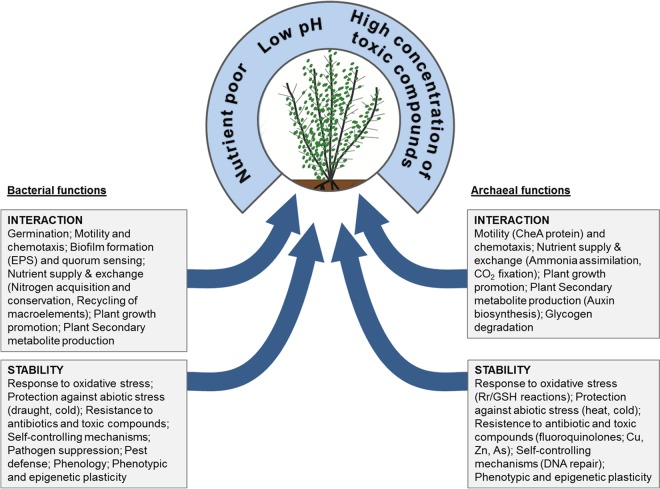
Model of *Archaea* and *Bacteria* contributing to bog functioning. Functions grouped as interaction and stability of the archaeal and bacterial microbiomes were deduced from metagenomic sequences that were annotated using functional subsystems of the SEED database. The examples in parentheses are the most distinctive and differentially abundant genetic signatures. EPS, extracellular polysaccharides; GSH, glutathione; Rr, rubrerythrin.

Summarizing the results, we developed a model showing the contributions of bacteria and archaea to ecosystem functioning (Fig. 6). Both prokaryotic groups have the potential to interact with plants and are potentially able to protect their host against biotic and abiotic stresses. Moreover, they contribute to the stability of the ecosystem to a certain extent. *Archaea* are found to have the potential to be involved in (i) plant-microbe interaction, (ii) fungus-microbe interaction, (iii) nutrient supply and exchange, (iv) protection against abiotic (especially oxidative and osmotic) stress, and (v) plant secondary metabolite production.

## DISCUSSION

Results of this study suggest that it is necessary to integrate *Archaea* into the synergistic concept of host-microbe interaction and bog functioning, which was established for bacteria (25). We could show that *Archaea* are a substantial component of the plant microbiomes and are able to fulfill functions for the host as well as for the ecosystem. *Archaea* are known as “food and survival artists” and for their ability to adapt to chronic energy stress (2, 30, 31). Both facts were confirmed for plant-associated *Archaea*, which were identified as being involved in nutrient supply and exchange. Our novel findings also suggest that they are involved in protection against abiotic stress as well as growth promotion and interact with plants as well as fungi.

In general, plant roots and rhizosphere provide microniches for specific microbial colonization. The anoxic and oxygen-limited conditions allow colonization of *Archaea* in high abundances like methanogenic and ammonium-oxidizing *Archaea* (5, 6). As for bog vegetation, we found these high abundances of *Archaea* filling specific niches in alpine raised bogs. Especially on bog vegetation forming lignified parts like plants of the classes Monocotyledons and Eudicotyledons, such as Eriophorum vaginatum and Vaccinium oxycoccus, respectively, high archaeal abundances were detected. Furthermore, PCoA clustering of 16S rRNA gene amplicons supported our observations of plant-specific colonization of *Archaea*. Moreover, the results are in accordance with previous findings showing the influence of changes in vegetation structure on the structure of methanogenic archaeal community in peatlands (32). Further, these bog plants were forming deep roots entering a special zone of the bog, the catotelm, which is characterized by anoxic and stable environmental conditions. As *Archaea* are more affected by abiotic than by biotic factors (7, 30), these stable and O_2_-free conditions in connection with the plant type might have the greatest influence on archaeal colonization. Similar to rice roots, where mainly *Euryarchaeota* were colonizing the roots (33, 34), the archaeal community of bog vegetation consisted particularly of *Euryarchaeota*, more precisely *Methanosarcina* and *Methanoregula* species revealed by the metagenomic data set. Signatures belonging to *Crenarchaeota*, *Thaumarchaeota*, *Korarchaeota*, and *Nanoarchaeota* were rare. Overall, the diversity in the 16S rRNA gene data set was low compared to the whole metagenomics shotgun-sequencing approach, similar to observations in other motive-based studies of bog ecosystems such as 16S rRNA gene studies of methanogens in boreal peats (35). This issue of greater identification of phyla and genera among the metagenomic sequences compared to the 16S rRNA sequences is known to be due to the databases used and biases in PCR amplification and amplicon sequencing (36). In general, the resolution of current databases for *Archaea* is limited on the genus level, for which reason additional phylogenetic analyses possess the potential to improve the taxonomical classification by assignment of OTUs against a DNA-based distance matrix. In our study, we found a distinct branch of OTUs belonging to *Thaumarchaeota*, which could not be assigned to classified *Thaumarchaeota* species, based on the RefSeq database. These OTUs might represent bog plant-specific, so far unclassified *Archaea*. Comparatively, the taxonomic structure of the metagenomic and 16S rRNA amplicon data sets showed high homogeneity on the phylum level, whereas the abundances of most dominant taxa on the genus level differed.

Bog ecosystems provide stable but extreme environmental conditions for all (micro)organisms. The interaction of bacteria and the bog vegetation supports the survival of both partners (25). In our study, we found functional signatures of *Archaea* indicating a so far unknown interaction with plants and their importance for the bog ecosystem itself. First, plants in general and especially plants inhabiting open bog ecosystems are highly affected by oxidative stress (37). We found functional signatures involved in the response to oxidative stress for plant-associated *Archaea*, which are known to be evolutionarily adapted to energy stress (30). This adaptation might enable the plant colonization under such extreme conditions and thereby indirectly supporting plant growth by the archaeal capability of nutrient fixation, similar to what was previously shown for bacteria colonizing sphagna (25). Interestingly, the level of genetic attributes encoding general and oxidative stress responses was high, which can be explained by the extreme conditions in the ecosystem. In contrast, osmotic stress response indicates drought stress associated with climate change. Second, we found archaeal functions for direct interaction with the plant. In the metagenomes of the bog vegetation, we detected functional signatures of *Archaea* involved in auxin biosynthesis (Table S2). A more detailed analysis with blastx revealed that the auxin biosynthesis genes coding for anthranilate phosphoribosyl-transferase, phosphoribosylanthranilate isomerase, and tryptophan synthase alpha and beta chains (EC 2.4.2.18, EC 5.3.1.24, and EC 4.2.1.20, respectively) are widely distributed among archaeal strains as they are also involved in the biosynthesis of amino acids (38). Whereas the gene encoding monoamine oxidase (EC 1.4.3.4), which is directly involved in auxin biosynthesis (39), was exclusively found in some archaeal species of the phylum *Euryarchaeota*. In this study, *Euryarchaeota* dominated the plant-associated archaeome on bog vegetation. Including the previous finding of the occurrence of the auxin biosynthesis genes, this might indicate that especially *Archaea* of the phylum *Euryarchaeota* adapted to the plant hosts. So far, auxin is known as a phytohormone regulating growth processes of the plant, and furthermore, it has been shown to be produced by bacteria (40); this is the first indication of direct interaction of archaeal strains with plants via auxin.

In addition to new aspects of the interaction with plants, we found functional signatures allowing interactions with other organisms (e.g., fungi), as well as relevance for the functioning of the entire bog ecosystem to a certain extent. The detected abundance of archaeal functions for glycogen degradation (Table S2) might give an explanation for previous observations of high abundances of *Archaea* in the mycorrhizosphere (41). Glycogen is a main storage unit of fungi and a part of fungal exudates, which support archaeal colonization (11). With respect to the entire ecosystem, *Archaea* associated with bog vegetation showed a high abundance in metagenome-derived functions associated with CO_2_ fixation and ammonium assimilation and thereby present in nutrient cycling of the bog. This is of great importance for the plants in a nutrient-poor environment, such as an ombrotrophic bog. Furthermore, the high number of attributes involved in CO_2_ fixation with regard to the main detected archaeal taxa *Methanomicrobia* and *Halobacteriaceae* is supported by previous work on the CO_2_ fixation capacity of *Euryarchaeota* (23).

### Conclusions.

*Archaea* are still an underdetected and little-studied part of the plant microbiome, and their contributions to health or disease remain mostly unknown. Our data provide a first evidence of the importance of *Archaea* as a functional component of the plant microbiome. Under the harsh environmental conditions of the bog ecosystem, *Archaea* contribute to the functioning of the ecosystem and vegetation by performing functions involved in nutrient cycling, stress response, and phytohormone biosynthesis and by interacting with both bacteria and their hosts. These archaeal properties should be further taken into account for microbiome-based treatment of plants in agriculture, especially in sites with extreme conditions, like rice fields and permanent agriculture. More efforts are needed to cultivate plant-associated archaea and to learn more about plant-associated archaeal diversity. Thus, before *Archaea* become part of the “disappearing microbiota” (42), we should at least know if we are going to miss them when they are gone.

## MATERIALS AND METHODS

### Experimental design and sampling procedure.

A microbiome-based analysis of the indigenous alpine peat bog vegetation in northern Styria, Austria, in November 2012 within two geographically distinct peat bogs, Rotmoos and Pürgschachen Moor, was conducted as described earlier (21). The plots for Rotmoos are located as follows: plot 1, N47 41.029 E15 09.284, 695 m; and plot 2, N47 41.059 E15 09.269, 695 m. The plots for Pürgschachen Moor are located as follows: plot 3, N47 34.835 E14 20.390, 632 m; and plot 4, N47 34.815 E14 20.482, 632 m. Both bogs have the typical structure of these ecosystems with an ombrotrophic, strongly acidic, large central part indicated by the dominance of different species of peat mosses (e.g., *Sphagnum magellanicum* and S. fuscum). In order to cover these typical ecological conditions, we selected randomly four plots (1 m^2^) dominated by S. magellanicum Brid. (section *Sphagnum*) in both locations. Frequent accompanied species were Sphagnum fuscum, S. angustifolium, S. capillifolium, Eriophorum vaginatum, Vaccinium myrtillus, and V. oxycoccus. In total, 46 samples of higher plants, bryophytes, and lichens with a minimum required fresh biomass of 10 g per sample were collected from the four selected plots, representing exemplary species naturally growing there. Samples were taken from the oxic catotelm layer and comprise for mosses and lichens the whole organisms and for vascular plants leaves and mainly roots. Our plant-ecology-based strategy focuses on the green and aerobic *Sphagnum*-layer only because the anaerobic part is less important for plant growth. Samples were stored separately in sterile plastic bags and transported on ice to the laboratory. All samples (*n =* 46) were subjected to 16S rRNA gene sequencing; in addition, 12 representative samples of the most frequently occurring species of bryophytes, vascular plants, and lichens were metagenome shotgun sequenced (Table 1).

### Isolation of total community DNA.

For DNA isolation, 5 g of sample material was physically disrupted with a sterile pestle and mortar and resuspended in 10 ml of 0.85% NaCl. Total community DNA was extracted from 2-ml aliquots after centrifugation (16,750 × *g* for 20 min at 4°C) using the FastDNA Spin kit for soil (MP Biomedical, Solon, OH). In a deviation from the manufacturer’s protocol, pellets were homogenized twice in a FastPrep FP120 instrument (Qbiogene, Inc., Bio 101, Carlsbad, CA) for 30 s at speed 5.0 m·s^−1^.

### Illumina sequencing and bioinformatics processing of 16S rRNA gene amplicons.

Microbial diversity was investigated targeting the V4 region with the primer pair 515F/806R (43) of the 16S rRNA gene using an Illumina MiSeq v2 platform (LGC genomics, Berlin, Germany). The PCR was conducted in triplicates, purified with the Wizard SV Gel and PCR cleanup system (Promega, Madison, WI), and pooled in equimolar concentrations prior to sequencing. The generated 16S rRNA gene Illumina libraries were subjected to standardized initial quality processing by the sequencing company (LGC genomics, Berlin, Germany). The open source software package Quantitative Insights into Microbial Ecology (QIIME) version 1.8 (44) was used to analyze the reads. At first, the data set was length and quality filtered to remove low-quality sequences, sequences that contained ambiguous characters and homopolymers, and chimeric sequences. The sequences were then clustered into operational taxonomic units (OTUs) with a 97% similarity cutoff (45) with the pick_open_reference_otus.py script using the USEARCH algorithm v6.1544 against the SILVA reference data set version 128 (46). Representative sequences for each OTU were taxonomically assigned using the UCLUST-based consensus classifier with default settings (47). OTUs that were classified to the *Archaea* phylum were filtered from the OTU table and normalized to 1,000 sequences per sample. α and β diversity indices, including rarefaction analysis, observed OTUs, Shannon diversity, Chao1 diversity estimation, and coverage were calculated. Two-dimensional (2D) PCoA plots based on jackknifed β diversity were calculated using weighted UniFrac indices and multiple resampling (1,000 sequences ×100 times) (48). Statistical analyses were done using an Adonis test with 999 permutations. A DNA distance maximum likelihood phylogenetic tree was constructed using the software package Phylip (49). Representative sequences of the archaeal OTUs from the 16S rRNA gene data set were aligned with the complete 16S rRNA gene reference sequence database (RefSeq, NCBI, release 82) prefiltered for *Archaea* using ClustalX version 2.1 (50) and MEGA version 7.0 (51). The maximum likelihood tree was visualized and modified using the interactive Tree of Life platform (iTOL, version 3) (52).

### Illumina metagenome sequencing and bioinformatics analysis.

Selected total-community DNA samples of the bog vegetation (Table S1) were sent for sequencing to Eurofins MWG Operon (Ebersberg, Germany; http://www.eurofinsgenomics.eu/). The sequencing was performed with an Illumina HiSeq 2500 system. Prior to sequencing, samples of the same species (bryophytes, vascular plants, and lichens) but from different locations were pooled (Table 1; Table S1). Samples MS1.9 and MS4.3, MS1.7 and MS4.4, MS1.1 and MS4.8, MS1.11 and MS4.13, MS1.4 and MS4.1, and MS1.8 and MS4.9 were pooled in equimolar ratios, respectively. The single samples MS1.10, MS1.2, MS1.3, MS1.5, MS1.6, and MS4.5 were pooled in equimolar ratios with the combined samples and sent for sequencing. The functional composition of the microbiome was analyzed using the metagenomic RAST (MG-RAST) server (53). For this purpose, the complete metagenomes were uploaded to the server and initially processed with default parameters filtered for artificial replicate sequences (54), low-quality (61) and short sequences, and sequences containing ambiguous bases. The annotation was done using hierarchical classification with the following default parameters: SEED subsystems as an annotation source, a maximum E value of 10^−5^, a minimum identity of 60%, and a minimum alignment length of 15 measured in amino acids for protein and base pairs for RNA databases. Within the annotated metagenomes, each single subsystem represented a group of sequences that encode a specific biological process or structural complex as defined by Overbeck et al. (55). The metagenomes were screened for functions annotated to *Archaea* within MG-RAST, and functional hits were subsequently exported for further analysis. The functional hits of each metagenome were normalized to the lowest number of sequences containing predicted proteins with known function (6,785,276). The structure and abundance of the functional subsystems were visualized using metagenome ANalyzer5 (MEGAN) (56) and in the latter compared with the relative distribution of bacterial functions. For further analysis of the distribution of the archaeal genes among the domain *Archaea*, blastx analysis was conducted (57). The taxonomic structure of the archaeal community was aligned and annotated with the RefSeq database as a reference (58). The taxonomic structure was then exported via the MG-RAST API server (59) and further visualized using krona tool version 2.7 (60).

10.1128/mSphere.00122-18.2 TABLE S1  List and composition of all 12 metagenomes uploaded and processed at the MG-RAST server. Metagenome sequences were obtained from total-community DNA from bog vegetation samples: Pleurozium schreberi, Sphagnum angustifolium, Vaccinium myrtilus, Calluna vulgaris, V. oxycoccus, Pinus mugo, S. fuscum, Andromeda polifolia, S. magellanicum, Polytrichum strictum, Eriophorum vaginatum, and Mylia anomala. Relative abundances are calculated from the total number of hits, whereas the relative abundance of *Archaea* within the prokaryotic fraction is shown as well in parentheses with an asterisk. Download TABLE S1, DOCX file, 0.1 MB. Copyright © 2018 Taffner et al. 2018 Taffner et al. This content is distributed under the terms of the Creative Commons Attribution 4.0 International license.

10.1128/mSphere.00122-18.3 TABLE S2  List of functional signatures involved in response to oxidative and osmotic stresses, glycogen metabolism, DNA repair, CO_2_ and nitrogen fixation, and secondary metabolite production of archaeal communities associated with bog vegetation. Functional signatures were obtained from 12 metagenomes, annotated using functional subsystems of the SEED database, and processed with MG-RAST. The according total abundance of each signature is given per sample. Download TABLE S2, DOCX file, 0.1 MB. Copyright © 2018 Taffner et al. 2018 Taffner et al. This content is distributed under the terms of the Creative Commons Attribution 4.0 International license.

10.1128/mSphere.00122-18.4 TABLE S3  Absolute and relative abundances of functional signatures of archaeal communities associated with bog vegetation as discussed in this study. Download TABLE S3, DOCX file, 0.01 MB. Copyright © 2018 Taffner et al. 2018 Taffner et al. This content is distributed under the terms of the Creative Commons Attribution 4.0 International license.

### Data availability.

The 16S rRNA Illumina libraries obtained from the sequencing company were deposited in the European Nucleotide Archive (ENA) under project no. PRJEB8670 accession no. ERS667879 to ERS667924 and ERS668032 to ERS668033. The complete OTU table was deposited in the Dryad Digital Repository under the accession identifier doi:10.5061/dryad.8n2d5. The complete metagenomes of Polytrichum strictum (4550991.3), Pleurozium schreberi (4550992.3), Sphagnum angustifolium (4550993.3), Vaccinium myrtillus (4550994.3), S. fuscum (4550995.3), S. magellanicum (4550996.3), Eriophorum vaginatum (4551107.3), Calluna vulgaris (4551108.3), V. oxycoccos (4551109.3), Pinus mugo (4551110.3), Andromeda polifolia (4551111.3), and Mylia anomala (4551112.3) are publicly available at the MG-Rast server (https://www.mg-rast.org/linkin.cgi?project=mgp7657) under the corresponding accession numbers.

## References

[1] Leininger S, Urich T, Schloter M, Schwark L, Qi J, Nicol GW, Prosser JI, Schuster SC, Schleper C 2006 *Archaea* predominate among ammonia-oxidizing prokaryotes in soils. Nature 442:806–809. doi:10.1038/nature04983. 16915287

[2] Adam PS, Borrel G, Brochier-Armanet C, Gribaldo S 2017 The growing tree of *Archaea*: new perspectives on their diversity, evolution and ecology. ISME J 11:2407–2425. doi:10.1038/ismej.2017.122. 28777382 PMC5649171

[3] Probst AJ, Auerbach AK, Moissl-Eichinger C 2013 *Archaea* on human skin. PLoS One 8:e65388. doi:10.1371/journal.pone.0065388. 23776475 PMC3680501

[4] Moissl-Eichinger C, Pausan M, Taffner J, Berg G, Bang C, Schmitz RA 2018 *Archaea* are interactive components of complex microbiomes. Trends Microbiol 26:70–85. doi:10.1016/j.tim.2017.07.004. 28826642

[5] Chelius MK, Triplett EW 2001 The diversity of archaea and bacteria in association with the roots of *Zea mays* L. Microb Ecol 41:252–263. doi:10.1007/s002480000087. 11391463

[6] Herrmann M, Saunders AM, Schramm A 2008 *Archaea* dominate the ammonia-oxidizing community in the rhizosphere of the freshwater macrophyte *Littorella uniflora*. Appl Environ Microbiol 74:3279–3283. doi:10.1128/AEM.02802-07. 18344332 PMC2394948

[7] Buée M, De Boer W, Martin F, van Overbeek L, Jurkevitch E 2009 The rhizosphere zoo: an overview of plant-associated communities of microorganisms, including phages, bacteria, archaea and of some of their structuring factors. Plant Soil 321:189–212. doi:10.1007/s11104-009-9991-3.

[8] Berendsen RL, Pieterse CMJ, Bakker PAHM 2012 The rhizosphere microbiome and plant health. Trends Plant Sci 17:478–486. doi:10.1016/j.tplants.2012.04.001. 22564542

[9] Knief C, Delmotte N, Chaffron S, Stark M, Innerebner G, Wassmann R, von Mering C, Vorholt JA 2012 Metaproteogenomic analysis of microbial communities in the phyllosphere and rhizosphere of rice. ISME J 6:1378–1390. doi:10.1038/ismej.2011.192. 22189496 PMC3379629

[10] Müller H, Berg C, Landa BB, Auerbach A, Moissl-Eichinger C, Berg G 2015 Plant genotype-specific archaeal and bacterial endophytes but similar *Bacillus* antagonists colonize Mediterranean olive trees. Front Microbiol 6:138. doi:10.3389/fmicb.2015.00138. 25784898 PMC4347506

[11] Karlsson AE, Johansson T, Bengtson P 2012 Archaeal abundance in relation to root and fungal exudation rates. FEMS Microbiol Ecol 80:305–311. doi:10.1111/j.1574-6941.2012.01298.x. 22611550

[12] Pump J, Pratscher J, Conrad R 2015 Colonization of rice roots with methanogenic archaea controls photosynthesis-derived methane emission. Environ Microbiol 17:2254–2260. doi:10.1111/1462-2920.12675. 25367104

[13] Berg G, Smalla K 2009 Plant species and soil type cooperatively shape the structure and function of microbial communities in the rhizosphere. FEMS Microbiol Ecol 68:1–13. doi:10.1111/j.1574-6941.2009.00654.x. 19243436

[14] Wagner MR, Lundberg DS, Del Rio TG, Tringe SG, Dangl JL, Mitchell-Olds T 2016 Host genotype and age shape the leaf and root microbiomes of a wild perennial plant. Nat Commun 7:12151. doi:10.1038/ncomms12151. 27402057 PMC4945892

[15] Yeoh YK, Dennis PG, Paungfoo-Lonhienne C, Weber L, Brackin R, Ragan MA, Schmidt S, Hugenholtz P 2017 Evolutionary conservation of a core root microbiome across plant phyla along a tropical soil chronosequence. Nat Commun 8:215. doi:10.1038/s41467-017-00262-8. 28790312 PMC5548757

[16] Bulgarelli D, Schlaeppi K, Spaepen S, Ver Loren van Themaat EVL, Schulze-Lefert P 2013 Structure and functions of the bacterial microbiota of plants. Annu Rev Plant Biol 64:807–838. doi:10.1146/annurev-arplant-050312-120106. 23373698

[17] Berg G, Rybakova D, Grube M, Köberl M 2016 The plant microbiome explored: implications for experimental botany. J Exp Bot 67:995–1002. doi:10.1093/jxb/erv466. 26547794 PMC5395086

[18] Opelt K, Berg C, Schönmann S, Eberl L, Berg G 2007 High specificity but contrasting biodiversity of *Sphagnum*-associated bacterial and plant communities in bog ecosystems independent of the geographical region. ISME J 1:502–516. doi:10.1038/ismej.2007.58. 18043652

[19] Opelt K, Chobot V, Hadacek F, Schönmann S, Eberl L, Berg G 2007 Investigations of the structure and function of bacterial communities associated with *Sphagnum* mosses. Environ Microbiol 9:2795–2809. doi:10.1111/j.1462-2920.2007.01391.x. 17922763

[20] Bragina A, Berg C, Cardinale M, Shcherbakov A, Chebotar V, Berg G 2012 *Sphagnum* mosses harbour highly specific bacterial diversity during their whole lifecycle. ISME J 6:802–813. doi:10.1038/ismej.2011.151. 22094342 PMC3309359

[21] Bragina A, Berg C, Berg G 2015 The core microbiome bonds the Alpine bog vegetation to a transkingdom metacommunity. Mol Ecol 24:4795–4807. doi:10.1111/mec.13342. 26335913

[22] Succow M, Joosten H 2001 Landschaftsökologische Moorkunde. Schweizerbart’sche Verlagsbuchhandlung, Stuttgart, Germany.

[23] Raghoebarsing AA, Smolders AJP, Schmid MC, Rijpstra WIC, Wolters-Arts M, Derksen J, Jetten MSM, Schouten S, Sinninghe Damsté JS, Lamers LPM, Roelofs JGM, Op den Camp HJM, Strous M 2005 Methanotrophic symbionts provide carbon for photosynthesis in peat bogs. Nature 436:1153–1156. doi:10.1038/nature03802. 16121180

[24] Dise NB 2009 Environmental science. Peatland response to global change. Science 326:810–811. doi:10.1126/science.1174268. 19892972

[25] Bragina A, Oberauner-Wappis L, Zachow C, Halwachs B, Thallinger GG, Müller H, Berg G 2014 The *Sphagnum* microbiome supports bog ecosystem functioning under extreme conditions. Mol Ecol 23:4498–4510. doi:10.1111/mec.12885. 25113243

[26] Clymo RS, Turunen J, Tolonen K 1998 Carbon accumulation in peatland. Oikos 81:368. doi:10.2307/3547057.

[27] Larmola T, Leppänen SM, Tuittila ES, Aarva M, Merilä P, Fritze H, Tiirola M 2014 Methanotrophy induces nitrogen fixation during peatland development. Proc Natl Acad Sci U S A 111:734–739. doi:10.1073/pnas.1314284111. 24379382 PMC3896166

[28] Bragina A, Cardinale M, Berg C, Berg G 2013 Vertical transmission explains the specific *Burkholderia* pattern in *Sphagnum* mosses at multi-geographic scale. Front Microbiol 4:394. doi:10.3389/fmicb.2013.00394. 24391630 PMC3866706

[29] Francis CA, Beman JM, Kuypers MMM 2007 New processes and players in the nitrogen cycle: the microbial ecology of anaerobic and archaeal ammonia oxidation. ISME J 1:19–27. doi:10.1038/ismej.2007.8. 18043610

[30] Valentine DL 2007 Adaptations to energy stress dictate the ecology and evolution of the *Archaea*. Nat Rev Microbiol 5:316–323. doi:10.1038/nrmicro1619. 17334387

[31] Cavicchioli R 2011 *Archaea*—timeline of the third domain. Nat Rev Microbiol 9:51–61. doi:10.1038/nrmicro2482. 21132019

[32] Rooney-Varga JN, Giewat MW, Duddleston KN, Chanton JP, Hines ME 2007 Links between archaeal community structure, vegetation type and methanogenic pathway in Alaskan peatlands. FEMS Microbiol Ecol 60:240–251. doi:10.1111/j.1574-6941.2007.00278.x. 17316328

[33] Conrad R, Klose M, Noll M, Kemnitz D, Bodelier PLE 2008 Soil type links microbial colonization of rice roots to methane emission. Glob Chang Biol 14:657–669. doi:10.1111/j.1365-2486.2007.01516.x.

[34] Edwards J, Johnson C, Santos-Medellín C, Lurie E, Podishetty NK, Bhatnagar S, Eisen JA, Sundaresan V 2015 Structure, variation, and assembly of the root-associated microbiomes of rice. Proc Natl Acad Sci U S A 112:E911–E920. doi:10.1073/pnas.1414592112. 25605935 PMC4345613

[35] Galand PE, Fritze H, Conrad R, Yrjälä K 2005 Pathways for methanogenesis and diversity of methanogenic archaea in three boreal peatland ecosystems. Appl Environ Microbiol 71:2195–2198. doi:10.1128/AEM.71.4.2195-2198.2005. 15812059 PMC1082526

[36] Poretsky R, Rodriguez-R LM, Luo C, Tsementzi D, Konstantinidis KT 2014 Strengths and limitations of 16S rRNA gene amplicon sequencing in revealing temporal microbial community dynamics. PLoS One 9:e93827. doi:10.1371/journal.pone.0093827. 24714158 PMC3979728

[37] Daniels R, Eddy A 1985 Handbook of European sphagna. Institute of Terrestrial Ecology, Natural Environmental Research Council, Cambrian News, Aberystwyth, United Kingdom.

[38] Gory Vert G, Walcher C, Chory J, Nemhauser J 2008 Integration of auxin and brassinosteroid pathways by auxin response factor 2. Proc Natl Acad Sci U S A 28:9829–9834. 10.1073/pnas.0803996105 PMC2474533 18599455

[39] Gaweska HM, Taylor AB, Hart PJ, Fitzpatrick PF 2013 The structure of the flavoprotein tryptophan-2-monooxygenase, a key enzyme in the formation of galls in plants. Biochemistry 52:2620–2626. doi:10.1021/bi4001563. 23521653 PMC3635830

[40] Spaepen S, Vanderleyden J 2011 Auxin and plant-microbe interactions. Cold Spring Harb Perspect 3:a001438. doi:10.1101/cshperspect.a001438. PMC3062209 21084388

[41] Bomberg M, Jurgens G, Saano A, Sen R, Timonen S 2003 Nested PCR detection of *Archaea* in defined compartments of pine mycorrhizospheres developed in boreal forest humus microcosms. FEMS Microbiol Ecol 43:163–171. doi:10.1111/j.1574-6941.2003.tb01055.x. 19719676

[42] Blaser MJ 2017 The theory of disappearing microbiota and the epidemics of chronic diseases. Nat Rev Immunol 17:461–463. doi:10.1038/nri.2017.77. 28749457

[43] Caporaso JG, Lauber CL, Walters WA, Berg-Lyons D, Lozupone CA, Turnbaugh PJ, Fierer N, Knight R 2011 Global patterns of 16S rRNA diversity at a depth of millions of sequences per sample. Proc Natl Acad Sci U S A 108(Suppl 1):4516–4522. doi:10.1073/pnas.1000080107. 20534432 PMC3063599

[44] Caporaso JG, Kuczynski J, Stombaugh J, Bittinger K, Bushman FD, Costello EK, Fierer N, Peña AG, Goodrich JK, Gordon JI, Huttley GA, Kelley ST, Knights D, Koenig JE, Ley RE, Lozupone CA, McDonald D, Muegge BD, Pirrung M, Reeder J, Sevinsky JR, Turnbaugh PJ, Walters WA, Widmann J, Yatsunenko T, Zaneveld J, Knight R 2010 QIIME allows analysis of high-throughput community sequencing data. Nat Methods 7:335–336. doi:10.1038/nmeth.f.303. 20383131 PMC3156573

[45] Schloss PD, Handelsman J 2006 Toward a census of bacteria in soil. PLoS Comput Biol 2:e92. doi:10.1371/journal.pcbi.0020092. 16848637 PMC1513271

[46] Quast CT, Pruesse E, Yilmaz P, Gerken J, Schweer T, Yarza P, Peplies J, Glöckner FO 2013 The SILVA ribosomal RNA gene database project: improved data processing and web-based tools. Nucleic Acids Res 41:D590–D596. doi:10.1093/nar/gks1219. 23193283 PMC3531112

[47] Edgar RC 2010 Search and clustering orders of magnitude faster than BLAST. Bioinformatics 26:2460–2461. doi:10.1093/bioinformatics/btq461. 20709691

[48] Lozupone C, Lladser ME, Knights D, Stombaugh J, Knight R 2011 UniFrac: an effective distance metric for microbial community comparison. ISME J 5:169–172. doi:10.1038/ismej.2010.133. 20827291 PMC3105689

[49] Felsenstein J 1989 Mathematics vs. evolution: mathematical evolutionary theory. Science 246:941–942. doi:10.1126/science.246.4932.941. 17812579

[50] Larkin MA, Blackshields G, Brown NP, Chenna R, McGettigan PA, McWilliam H, Valentin F, Wallace IM, Wilm A, Lopez R, Thompson JD, Gibson TJ, Higgins DG 2007 Clustal W and Clustal X version 2.0. Bioinformatics 23:2947–2948. doi:10.1093/bioinformatics/btm404. 17846036

[51] Kumar S, Stecher G, Tamura K 2016 MEGA7: Molecular Evolutionary Genetics Analysis version 7.0 for bigger datasets. Mol Biol Evol 33:1870–1874. doi:10.1093/molbev/msw054. 27004904 PMC8210823

[52] Letunic I, Bork P 2016 Interactive Tree of Life (iTOL) v3: an online tool for the display and annotation of phylogenetic and other trees. Nucleic Acids Res 44:W242–WW245. doi:10.1093/nar/gkw290. 27095192 PMC4987883

[53] Meyer F, Paarmann D, D’Souza M, Olson R, Glass EM, Kubal M, Paczian T, Rodriguez A, Stevens R, Wilke A, Wilkening J, Edwards RA 2008 The metagenomics RAST server—a public resource for the automatic phylogenetic and functional analysis of metagenomes. BMC Bioinformatics 9:386. doi:10.1186/1471-2105-9-386. 18803844 PMC2563014

[54] Gomez-Alvarez V, Teal TK, Schmidt TM 2009 Systematic artifacts in metagenomes from complex microbial communities. ISME J 3:1314–1317. doi:10.1038/ismej.2009.72. 19587772

[55] Overbeek R, Begley T, Butler RM, Choudhuri JV, Chuang HY, Cohoon M, de Crécy-Lagard V, Diaz N, Disz T, Edwards R, Fonstein M, Frank ED, Gerdes S, Glass EM, Goesmann A, Hanson A, Iwata-Reuyl D, Jensen R, Jamshidi N, Krause L, Kubal M, Larsen N, Linke B, McHardy AC, Meyer F, Neuweger H, Olsen G, Olson R, Osterman A, Portnoy V, Pusch GD, Rodionov DA, Rückert C, Steiner J, Stevens R, Thiele I, Vassieva O, Ye Y, Zagnitko O, Vonstein V 2005 The subsystems approach to genome annotation and its use in the project to annotate 1,000 genomes. Nucleic Acids Res 33:5691–5702. doi:10.1093/nar/gki866. 16214803 PMC1251668

[56] Huson DH, Auch AF, Qi J, Schuster SC 2007 MEGAN analysis of metagenomic data. Genome Res 17:377–386. doi:10.1101/gr.5969107. 17255551 PMC1800929

[57] Altschul SF, Gish W, Miller W, Myers EW, Lipman DJ 1990 Basic local alignment search tool. J Mol Biol 215:403–410. doi:10.1016/S0022-2836(05)80360-2. 2231712

[58] Pruitt KD, Tatusova T, Maglott DR 2007 NCBI reference sequences (RefSeq): a curated non-redundant sequence database of genomes, transcripts and proteins. Nucleic Acids Res 35:D61–D65. doi:10.1093/nar/gkl842. 17130148 PMC1716718

[59] Wilke A, Bischof J, Harrison T, Brettin T, D’Souza M, Gerlach W, Matthews H, Paczian T, Wilkening J, Glass EM, Desai N, Meyer F 2015 A RESTful API for accessing microbial community data for MG-RAST. PLoS Comp Biol 11:e1004008. doi:10.1371/journal.pcbi.1004008. PMC4287624 25569221

[60] Ondov BD, Bergman NH, Phillippy AM 2011 Interactive metagenomic visualization in a Web browser. BMC Bioinformatics 12:385. doi:10.1186/1471-2105-12-385. 21961884 PMC3190407

[61] Cox MP, Peterson DA, Biggs PJ 2010 SolexaQA: at-a-glance quality assessment of Illumina second-generation sequencing data. BMC Bioinformatics 11:485. doi:10.1186/1471-2105-11-485. 20875133 PMC2956736

