# Supplementary figures and images for: What Is the Role of Archaea in Plants? New Insights from the Vegetation of Alpine Bogs
Source: mSphere. 2018 May 9;3(3):e00122-18. doi: 10.1128/mSphere.00122-18 (PMC5956146; doi:10.1128/mSphere.00122-18)

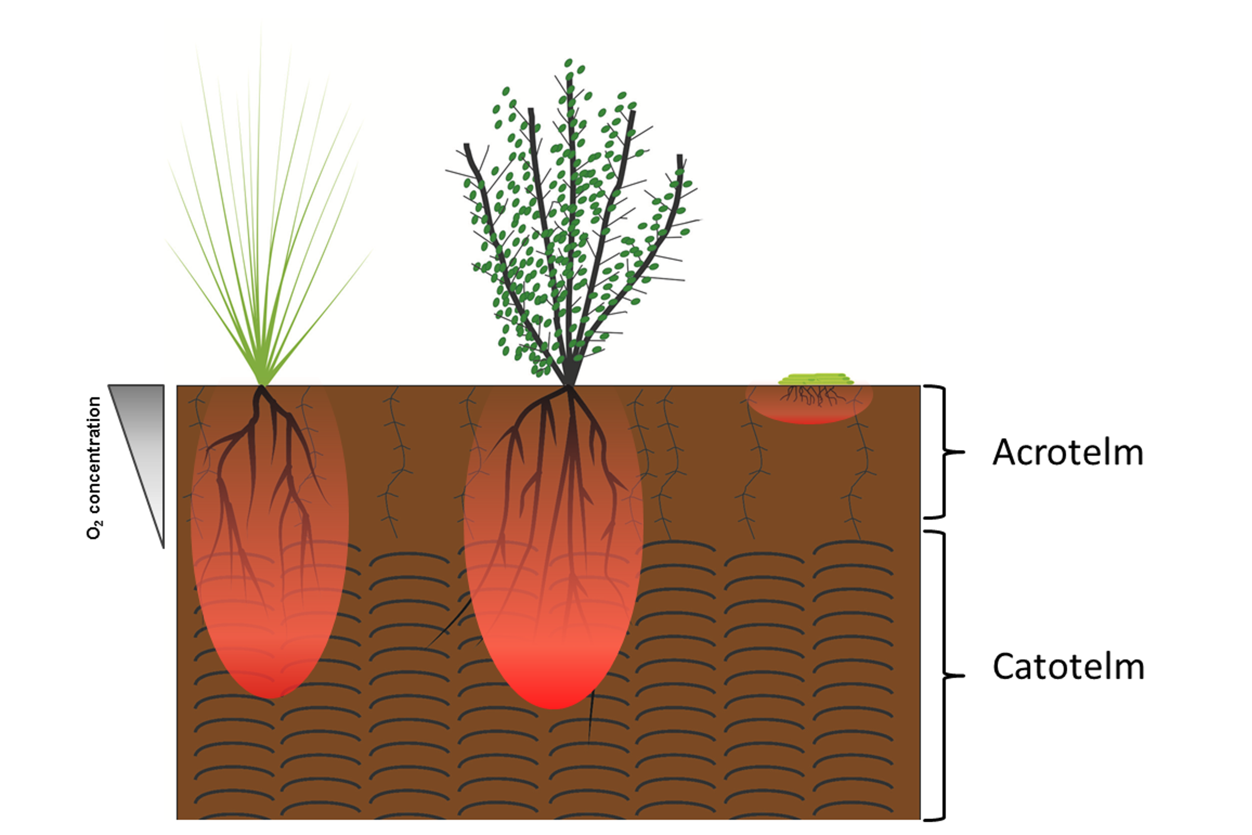

Supplement: FIG S1 [file sph003182536sf1.tif]
