# Supplementary material for: What Is the Role of Archaea in Plants? New Insights from the Vegetation of Alpine Bogs
Source: mSphere. 2018 May 9;3(3):e00122-18. doi: 10.1128/mSphere.00122-18 (PMC5956146; doi:10.1128/mSphere.00122-18)
Supplement: TABLE S1 [file sph003182536st1.docx]

| **MG-RAST ID** | **Sample ID** | **Species** | **Growth form** | **Family** | **Archaea** | **Archaeal seq.** | **Bacteria** | **Eukaryota** | **Viruses** | **Other** | **Total seq.** | **Total prokaryotic seq.** |
| --- | --- | --- | --- | --- | --- | --- | --- | --- | --- | --- | --- | --- |
| 4550991.3 | MS1.10 | *Polytrichum strictum* | Other mosses | *Polytrichaceae* | 0.43 (0.46*) | 84098 | 94.88 | 4.56 | 0.04 | 0.09 | 19372399 | 18465301 |
| 4550992.3 | MS1.1/4.8 | *Pleurozium schreberi* | Other mosses | *Hylocomiaceae* | 0.40 (0.44*) | 81696 | 91.53 | 7.98 | 0.04 | 0.05 | 20420373 | 18772985 |
| 4550993.3 | MS1.2 | *Sphagnum angustifolium* | Peat mosses | *Sphagnaceae* | 0.42 (0.45*) | 98987 | 93.39 | 6.03 | 0.06 | 0.10 | 23503959 | 22049262 |
| 4550994.3 | MS1.3 | *Vaccinium myrtillus* | Dwarf shrubs | *Ericaceae* | 0.17 (0.21*) | 27700 | 79.52 | 20.23 | 0.03 | 0.05 | 16498217 | 13147480 |
| 4550995.3 | MS1.7/4.4 | *Sphagnum fuscum* | Peat mosses | *Sphagnaceae* | 0.62 (0.66*) | 100860 | 92.51 | 6.66 | 0.05 | 0.15 | 16296987 | 15177794 |
| 4550996.3 | MS1.9/4.3 | *Sphagnum magellanicum* | Peat mosses | *Sphagnaceae* | 0.57 (0.61*) | 86472 | 92.20 | 6.99 | 0.07 | 0.17 | 15207119 | 14107365 |
| 4551107.3 | MS1.11/4.13 | *Eriophorum vaginatum* | Graminoids | *Cyperaceae* | 0.38 (0.41*) | 88249 | 92.17 | 7.34 | 0.04 | 0.07 | 23275000 | 21540827 |
| 4551108.3 | MS1.4/4.1 | *Calluna vulgaris* | Dwarf shrubs | *Ericaceae* | 0.34 (0.38*) | 65157 | 89.54 | 9.96 | 0.05 | 0.11 | 18973747 | 17054329 |
| 4551109.3 | MS1.5 | *Vaccinium oxycoccos* | Dwarf shrubs | *Ericaceae* | 0.28 (0.35*) | 39115 | 80.50 | 19.12 | 0.04 | 0.06 | 13946591 | 11266172 |
| 4551110.3 | MS1.6 | *Pinus mugo* | Coniferous trees | *Pinacaea* | 0.27 (0.43*) | 22363 | 63.86 | 35.73 | 0.06 | 0.08 | 8189022 | 5251945 |
| 4551111.3 | MS1.8/4.9 | *Andromeda polifolia* | Dwarf shrubs | *Ericaceae* | 0.30 (0.37*) | 48546 | 80.00 | 19.59 | 0.04 | 0.06 | 16451849 | 13210592 |
| 4551112.3 | MS4.5 | *Mylia anomala* | Liverworts | *Myliaceae* | 0.48 (0.51*) | 99509 | 93.43 | 5.96 | 0.05 | 0.08 | 20603933 | 19350593 |
