# Supplementary material for: What Is the Role of Archaea in Plants? New Insights from the Vegetation of Alpine Bogs
Source: mSphere. 2018 May 9;3(3):e00122-18. doi: 10.1128/mSphere.00122-18 (PMC5956146; doi:10.1128/mSphere.00122-18)
Supplement: TABLE S2 [file sph003182536st2.docx]

| **SEED L1** | **SEED L2** | **SEED L3** | **SEED L4** | **polytrichum strictum** | **pleurozium schreberi** | **sphagnum angustifolium** | **vaccinium myrtillus** | **sphagnum fuscum** | **sphagnum magellanicum** | **eriophorum vaginatum** | **calluna vulgaris** | **vaccinium oxycoccus** | **pinus mugo** | **andromeda polifolia** | **mylia anomala** |
| --- | --- | --- | --- | --- | --- | --- | --- | --- | --- | --- | --- | --- | --- | --- | --- |
| **total abundance of archaeal hits** | |  |  | 17468 | 16659 | 16802 | 4298 | 23051 | 22362 | 15393 | 14151 | 10392 | 5808 | 10755 | 18844 |
| **Secondary Metabolism** | **Plant Hormones** |  |  | 229 | 219 | 200 | 34 | 235 | 237 | 169 | 144 | 105 | 46 | 115 | 213 |
|  |  | Auxin_biosynthesis | Anthranilate phosphoribosyltransferase (EC 2.4.2.18) | 66 | 80 | 82 | 11 | 64 | 80 | 53 | 35 | 30 | 13 | 44 | 71 |
|  |  | Auxin_biosynthesis | Phosphoribosylanthranilate isomerase (EC 5.3.1.24) | 36 | 20 | 32 | 5 | 65 | 34 | 14 | 14 | 15 | 1 | 6 | 25 |
|  |  | Auxin_biosynthesis | Tryptophan synthase alpha chain (EC 4.2.1.20) | 34 | 15 | 6 | 1 | 18 | 31 | 23 | 24 | 8 | 4 | 7 | 5 |
|  |  | Auxin_biosynthesis | Tryptophan synthase beta chain (EC 4.2.1.20) | 89 | 100 | 72 | 17 | 88 | 87 | 75 | 67 | 49 | 28 | 48 | 108 |
| **Nitrogen Metabolism** | **Nitrogen fixation** |  |  | 1 | 1 | 0 | 0 | 1 | 7 | 2 | 1 | 6 | 0 | 0 | 1 |
|  |  | Homocitrate synthase (EC 2.3.3.14) |  | 1 | 0 | 0 | 0 | 1 | 6 | 1 | 0 | 0 | 0 | 0 | 1 |
|  |  | Nitrogenase (iron-iron) beta chain (EC 1.18.6.1) |  | 0 | 0 | 0 | 0 | 0 | 0 | 1 | 0 | 1 | 0 | 0 | 0 |
|  |  | Nitrogenase (molybdenum-iron) reductase |  | 0 | 1 | 0 | 0 | 0 | 0 | 0 | 0 | 0 | 0 | 0 | 0 |
|  |  | Nitrogenase (vanadium-iron) beta chain (EC 1.18.6.1) |  | 0 | 0 | 0 | 0 | 0 | 0 | 0 | 0 | 3 | 0 | 0 | 0 |
|  |  | Nitrogenase vanadium-cofactor synthesis protein VnfE |  | 0 | 0 | 0 | 0 | 0 | 0 | 0 | 0 | 1 | 0 | 0 | 0 |
|  |  | Nitrogenase vanadium-cofactor synthesis protein VnfN |  | 0 | 0 | 0 | 0 | 0 | 1 | 0 | 0 | 0 | 0 | 0 | 0 |
|  |  | Transcriptional repressor of nif and glnA operons |  | 0 | 0 | 0 | 0 | 0 | 0 | 0 | 1 | 1 | 0 | 0 | 0 |
| **Stress Response** | **Oxidative stress** |  |  | 192 | 223 | 162 | 58 | 270 | 275 | 214 | 198 | 161 | 85 | 150 | 269 |
|  |  | Oxidative_stress | Alkyl hydroperoxide reductase subunit C-like protein | 19 | 20 | 17 | 4 | 16 | 30 | 20 | 13 | 6 | 12 | 8 | 25 |
|  |  | Oxidative_stress | Catalase (EC 1.11.1.6) | 19 | 45 | 29 | 17 | 28 | 31 | 40 | 53 | 37 | 11 | 37 | 23 |
|  |  | Oxidative_stress | Manganese superoxide dismutase (EC 1.15.1.1) | 2 | 1 | 2 | 0 | 3 | 5 | 2 | 3 | 3 | 3 | 3 | 5 |
|  |  | Oxidative_stress | Phytochrome, two-component sensor histidine kinase (EC 2.7.3.-) | 12 | 10 | 12 | 0 | 21 | 15 | 9 | 8 | 6 | 6 | 1 | 14 |
|  |  | Oxidative_stress | Probable peroxiredoxin (EC 1.11.1.15) | 3 | 4 | 5 | 2 | 4 | 4 | 3 | 2 | 7 | 1 | 1 | 8 |
|  |  | Oxidative_stress | Rubrerythrin | 0 | 0 | 1 | 0 | 0 | 0 | 1 | 1 | 2 | 0 | 0 | 0 |
|  |  | Oxidative_stress | Superoxide dismutase [Cu-Zn] precursor (EC 1.15.1.1) | 0 | 1 | 1 | 0 | 0 | 0 | 1 | 2 | 0 | 1 | 1 | 0 |
|  |  | Oxidative_stress | Superoxide dismutase [Fe] (EC 1.15.1.1) | 1 | 1 | 1 | 0 | 4 | 0 | 1 | 2 | 3 | 0 | 1 | 1 |
|  |  | Oxidative_stress | Superoxide dismutase [Mn/Fe] (EC 1.15.1.1) | 0 | 0 | 0 | 0 | 3 | 0 | 0 | 2 | 3 | 0 | 2 | 1 |
|  |  | Oxidative_stress | Superoxide dismutase [Mn] (EC 1.15.1.1) | 0 | 1 | 2 | 0 | 2 | 0 | 3 | 0 | 0 | 0 | 0 | 2 |
|  |  | Oxidative_stress | Superoxide reductase (EC 1.15.1.2) | 0 | 0 | 0 | 0 | 0 | 0 | 0 | 0 | 1 | 0 | 0 | 0 |
|  |  | Rubrerythrin.1 | Rubredoxin | 4 | 1 | 0 | 1 | 0 | 2 | 1 | 1 | 2 | 0 | 3 | 1 |
|  |  | Rubrerythrin.1 | Fe-S oxidoreductase-like protein in Rubrerythrin cluster | 2 | 1 | 2 | 1 | 0 | 4 | 2 | 1 | 0 | 2 | 0 | 2 |
|  |  | Glutathione:_Biosynthesis_and_gamma-glutamyl_cycle | Gamma-glutamyltranspeptidase (EC 2.3.2.2) | 31 | 13 | 5 | 5 | 32 | 29 | 18 | 20 | 14 | 13 | 31 | 12 |
|  |  | Glutathione:_Biosynthesis_and_gamma-glutamyl_cycle | Glutamate--cysteine ligase (EC 6.3.2.2) | 0 | 1 | 0 | 0 | 0 | 0 | 0 | 0 | 0 | 0 | 0 | 0 |
|  |  | Glutathione:_Biosynthesis_and_gamma-glutamyl_cycle | Glutamate--cysteine ligase archaeal (EC 6.3.2.2) | 0 | 24 | 0 | 0 | 0 | 0 | 11 | 1 | 5 | 0 | 0 | 0 |
|  |  | Glutathione:_Biosynthesis_and_gamma-glutamyl_cycle | Similar to 5-oxoprolinase (EC 3.5.2.9) and Methylhydantoinases A, B (EC 3.5.2.14) | 62 | 56 | 62 | 18 | 95 | 93 | 62 | 51 | 46 | 33 | 41 | 127 |
|  |  | Redox-dependent_regulation_of_nucleus_processes | NAD-dependent glyceraldehyde-3-phosphate dehydrogenase (EC 1.2.1.12) | 2 | 0 | 1 | 1 | 0 | 1 | 0 | 0 | 0 | 0 | 0 | 0 |
|  |  | Redox-dependent_regulation_of_nucleus_processes | NAD-dependent protein deacetylase of SIR2 family | 17 | 15 | 12 | 4 | 32 | 30 | 19 | 19 | 14 | 1 | 9 | 34 |
|  |  | Redox-dependent_regulation_of_nucleus_processes | NADPH-dependent glyceraldehyde-3-phosphate dehydrogenase (EC 1.2.1.13) | 0 | 0 | 0 | 0 | 2 | 3 | 1 | 1 | 2 | 0 | 0 | 1 |
|  |  | Redox-dependent_regulation_of_nucleus_processes | Nicotinamidase (EC 3.5.1.19) | 7 | 7 | 2 | 1 | 11 | 11 | 9 | 6 | 3 | 1 | 4 | 8 |
|  |  | Redox-dependent_regulation_of_nucleus_processes | Nicotinate phosphoribosyltransferase (EC 2.4.2.11) | 0 | 2 | 1 | 2 | 6 | 4 | 0 | 1 | 0 | 0 | 1 | 0 |
|  |  | Glutathione:_Non-redox_reactions | Glutathione S-transferase, omega (EC 2.5.1.18) | 11 | 20 | 7 | 2 | 11 | 13 | 11 | 11 | 7 | 1 | 7 | 5 |
|  | **Osmotic stress** |  |  | 54 | 48 | 62 | 18 | 94 | 84 | 45 | 58 | 54 | 33 | 72 | 79 |
|  |  | Choline_and_Betaine_Uptake_and_Betaine_Biosynthesis | Choline-sulfatase (EC 3.1.6.6) | 13 | 16 | 18 | 1 | 23 | 23 | 9 | 4 | 22 | 15 | 31 | 20 |
|  |  | Choline_and_Betaine_Uptake_and_Betaine_Biosynthesis | Glycine betaine ABC transport system, ATP-binding protein OpuAA (EC 3.6.3.32) | 5 | 1 | 3 | 0 | 3 | 3 | 4 | 4 | 0 | 0 | 4 | 5 |
|  |  | Choline_and_Betaine_Uptake_and_Betaine_Biosynthesis | Glycine betaine ABC transport system, permease protein OpuAB | 0 | 0 | 0 | 0 | 0 | 0 | 0 | 1 | 0 | 1 | 1 | 0 |
|  |  | Choline_and_Betaine_Uptake_and_Betaine_Biosynthesis | L-proline glycine betaine ABC transport system permease protein ProV (TC 3.A.1.12.1) | 36 | 31 | 39 | 15 | 66 | 57 | 31 | 49 | 28 | 17 | 36 | 52 |
|  |  | Choline_and_Betaine_Uptake_and_Betaine_Biosynthesis | Osmotically activated L-carnitine/choline ABC transporter, ATP-binding protein OpuCA | 0 | 0 | 0 | 0 | 2 | 1 | 0 | 0 | 0 | 0 | 0 | 0 |
|  |  | Choline_and_Betaine_Uptake_and_Betaine_Biosynthesis | Sarcosine oxidase alpha subunit (EC 1.5.3.1) | 0 | 0 | 2 | 2 | 0 | 0 | 1 | 0 | 4 | 0 | 0 | 2 |
| SEED L1 | SEED L2 | SEED L3 | SEED L4 | polytrichum strictum | pleurozium schreberi | sphagnum angustifolium | vaccinium myrtillus | sphagnum fuscum | sphagnum magellanicum | eriophorum vaginatum | calluna vulgaris | vaccinium oxycoccus | pinus mugo | andromeda polifolia | mylia anomala |
| **Carbohydrates** | **CO_2_ fixation** |  |  | 194 | 207 | 177 | 49 | 307 | 268 | 179 | 178 | 100 | 73 | 134 | 203 |
|  |  | Calvin-Benson_cycle | Fructose-1,6-bisphosphatase, GlpX type (EC 3.1.3.11) | 0 | 0 | 0 | 1 | 0 | 0 | 1 | 0 | 0 | 0 | 0 | 4 |
|  |  | Calvin-Benson_cycle | NAD(P)-dependent glyceraldehyde 3-phosphate dehydrogenase archaeal (EC 1.2.1.59) | 0 | 0 | 0 | 0 | 0 | 0 | 0 | 0 | 2 | 0 | 0 | 0 |
|  |  | Calvin-Benson_cycle | NAD-dependent glyceraldehyde-3-phosphate dehydrogenase (EC 1.2.1.12) | 2 | 0 | 1 | 1 | 0 | 1 | 0 | 0 | 0 | 0 | 0 | 0 |
|  |  | Calvin-Benson_cycle | NADPH-dependent glyceraldehyde-3-phosphate dehydrogenase (EC 1.2.1.13) | 0 | 0 | 0 | 0 | 2 | 3 | 1 | 1 | 2 | 0 | 0 | 1 |
|  |  | Calvin-Benson_cycle | Phosphoglycerate kinase (EC 2.7.2.3) | 13 | 10 | 4 | 2 | 9 | 17 | 18 | 9 | 3 | 3 | 11 | 13 |
|  |  | Calvin-Benson_cycle | Ribose 5-phosphate isomerase A (EC 5.3.1.6) | 23 | 36 | 31 | 3 | 68 | 24 | 20 | 31 | 9 | 15 | 19 | 34 |
|  |  | Calvin-Benson_cycle | Ribulose-phosphate 3-epimerase (EC 5.1.3.1) | 5 | 3 | 4 | 3 | 8 | 3 | 4 | 1 | 0 | 0 | 1 | 7 |
|  |  | Calvin-Benson_cycle | Transketolase, C-terminal section (EC 2.2.1.1) | 1 | 4 | 2 | 1 | 12 | 10 | 8 | 2 | 2 | 1 | 2 | 6 |
|  |  | Calvin-Benson_cycle | Transketolase, N-terminal section (EC 2.2.1.1) | 7 | 8 | 9 | 1 | 11 | 10 | 5 | 16 | 0 | 0 | 8 | 4 |
|  |  | Calvin-Benson_cycle | Triosephosphate isomerase (EC 5.3.1.1) | 1 | 0 | 0 | 0 | 0 | 0 | 1 | 0 | 0 | 0 | 0 | 0 |
|  |  | Carboxysome | Carbonic anhydrase (EC 4.2.1.1) | 1 | 2 | 3 | 0 | 2 | 2 | 2 | 0 | 1 | 3 | 0 | 2 |
|  |  | Carboxysome | NADH dehydrogenase (EC 1.6.99.3) | 15 | 19 | 15 | 2 | 47 | 25 | 8 | 8 | 3 | 3 | 10 | 20 |
|  |  | CO2_uptake,_carboxysome | Hypothetical transmembrane protein coupled to NADH-ubiquinone oxidoreductase chain 5 homolog | 3 | 2 | 6 | 0 | 7 | 6 | 0 | 1 | 0 | 0 | 0 | 1 |
|  |  | Photorespiration_(oxidative_C2_cycle) | 2-hydroxy-3-oxopropionate reductase (EC 1.1.1.60) | 9 | 8 | 5 | 3 | 5 | 9 | 13 | 6 | 11 | 11 | 17 | 4 |
|  |  | Photorespiration_(oxidative_C2_cycle) | 2-oxoglutarate/malate translocator | 0 | 0 | 2 | 0 | 0 | 0 | 0 | 0 | 0 | 0 | 1 | 0 |
|  |  | Photorespiration_(oxidative_C2_cycle) | Aminomethyltransferase (glycine cleavage system T protein) (EC 2.1.2.10) | 3 | 1 | 4 | 1 | 4 | 6 | 3 | 1 | 0 | 0 | 1 | 5 |
|  |  | Photorespiration_(oxidative_C2_cycle) | Catalase (EC 1.11.1.6) | 19 | 45 | 29 | 17 | 28 | 31 | 40 | 53 | 37 | 11 | 37 | 23 |
|  |  | Photorespiration_(oxidative_C2_cycle) | Dihydrolipoamide dehydrogenase (EC 1.8.1.4) | 3 | 0 | 2 | 0 | 1 | 0 | 2 | 1 | 2 | 0 | 0 | 3 |
|  |  | Photorespiration_(oxidative_C2_cycle) | Glycine cleavage system H protein | 13 | 22 | 15 | 0 | 17 | 20 | 10 | 16 | 7 | 9 | 1 | 11 |
|  |  | Photorespiration_(oxidative_C2_cycle) | Glycine dehydrogenase [decarboxylating] (glycine cleavage system P1 protein) (EC 1.4.4.2) | 1 | 0 | 2 | 4 | 1 | 3 | 1 | 1 | 0 | 0 | 0 | 0 |
|  |  | Photorespiration_(oxidative_C2_cycle) | Glycine dehydrogenase [decarboxylating] (glycine cleavage system P2 protein) (EC 1.4.4.2) | 40 | 22 | 26 | 5 | 47 | 47 | 18 | 15 | 6 | 11 | 18 | 29 |
|  |  | Photorespiration_(oxidative_C2_cycle) | Glycolate dehydrogenase (EC 1.1.99.14), iron-sulfur subunit GlcF | 10 | 8 | 3 | 0 | 0 | 6 | 3 | 3 | 0 | 0 | 0 | 4 |
|  |  | Photorespiration_(oxidative_C2_cycle) | Glycolate dehydrogenase (EC 1.1.99.14), subunit GlcD | 3 | 3 | 2 | 1 | 12 | 0 | 1 | 0 | 0 | 4 | 0 | 9 |
|  |  | Photorespiration_(oxidative_C2_cycle) | Malate synthase (EC 2.3.3.9) | 0 | 0 | 0 | 0 | 0 | 1 | 0 | 0 | 0 | 0 | 0 | 0 |
|  |  | Photorespiration_(oxidative_C2_cycle) | Phosphoglycolate phosphatase (EC 3.1.3.18) | 0 | 0 | 0 | 0 | 0 | 3 | 0 | 0 | 0 | 0 | 0 | 0 |
|  |  | Photorespiration_(oxidative_C2_cycle) | Serine hydroxymethyltransferase (EC 2.1.2.1) | 21 | 12 | 11 | 4 | 24 | 37 | 15 | 12 | 12 | 2 | 7 | 21 |
|  |  | Photorespiration_(oxidative_C2_cycle) | Serine--glyoxylate aminotransferase (EC 2.6.1.45) | 1 | 2 | 1 | 0 | 2 | 4 | 5 | 1 | 3 | 0 | 1 | 2 |
|  | **Polysaccharides** | Glycogen_metabolism |  | 437 | 404 | 435 | 62 | 427 | 350 | 393 | 306 | 257 | 111 | 257 | 379 |
|  |  | Glycogen_metabolism | 1,4-alpha-glucan (glycogen) branching enzyme, GH-13-type (EC 2.4.1.18) | 13 | 16 | 25 | 4 | 32 | 35 | 16 | 11 | 13 | 6 | 15 | 34 |
|  |  | Glycogen_metabolism | 4-alpha-glucanotransferase (amylomaltase) (EC 2.4.1.25) | 21 | 24 | 14 | 4 | 13 | 10 | 13 | 13 | 13 | 3 | 12 | 16 |
|  |  | Glycogen_metabolism | Glycogen branching enzyme, GH-57-type, archaeal (EC 2.4.1.18) | 176 | 146 | 181 | 14 | 116 | 82 | 156 | 115 | 106 | 28 | 75 | 138 |
|  |  | Glycogen_metabolism | Glycogen debranching enzyme (EC 3.2.1.-) | 48 | 36 | 44 | 10 | 56 | 60 | 39 | 32 | 15 | 15 | 21 | 48 |
|  |  | Glycogen_metabolism | Glycogen phosphorylase (EC 2.4.1.1) | 137 | 126 | 112 | 19 | 126 | 99 | 115 | 71 | 76 | 40 | 104 | 95 |
|  |  | Glycogen_metabolism | Glycogen synthase, ADP-glucose transglucosylase (EC 2.4.1.21) | 0 | 0 | 1 | 1 | 0 | 0 | 1 | 0 | 0 | 1 | 0 | 0 |
|  |  | Glycogen_metabolism | Predicted glycogen synthase, ADP-glucose transglucosylase (EC 2.4.1.21), Actinobacterial type | 2 | 3 | 3 | 0 | 5 | 5 | 3 | 5 | 4 | 0 | 3 | 2 |
|  |  | Glycogen_metabolism | Putative glycogen debranching enzyme, archaeal type, TIGR01561 | 40 | 53 | 55 | 10 | 79 | 59 | 50 | 59 | 30 | 18 | 27 | 46 |
| SEED L1 | SEED L2 | SEED L3 | SEED L4 | polytrichum strictum | pleurozium schreberi | sphagnum angustifolium | vaccinium myrtillus | sphagnum fuscum | sphagnum magellanicum | eriophorum vaginatum | calluna vulgaris | vaccinium oxycoccus | pinus mugo | andromeda polifolia | mylia anomala |
| **DNA Metabolism** | **DNA repair** |  |  | 667 | 673 | 668 | 130 | 878 | 933 | 532 | 545 | 356 | 189 | 321 | 649 |
|  |  | DNA_repair,_bacterial_MutL-MutS_system | DNA mismatch repair protein MutL | 37 | 58 | 41 | 8 | 37 | 48 | 25 | 35 | 8 | 5 | 13 | 30 |
|  |  | DNA_repair,_bacterial_MutL-MutS_system | DNA mismatch repair protein MutS | 79 | 90 | 82 | 4 | 91 | 78 | 50 | 60 | 54 | 17 | 39 | 58 |
|  |  | DNA_repair,_bacterial_MutL-MutS_system | MutS domain protein, family 4 | 1 | 0 | 0 | 0 | 1 | 0 | 4 | 0 | 0 | 0 | 0 | 1 |
|  |  | DNA_repair,_bacterial | DNA repair protein RadC | 1 | 1 | 4 | 0 | 0 | 13 | 4 | 0 | 1 | 0 | 1 | 2 |
|  |  | DNA_repair,_bacterial | Exonuclease SbcC | 0 | 3 | 1 | 1 | 5 | 9 | 3 | 1 | 2 | 0 | 0 | 0 |
|  |  | DNA_repair,_bacterial | Exonuclease SbcD | 1 | 0 | 0 | 0 | 0 | 0 | 0 | 0 | 1 | 0 | 0 | 0 |
|  |  | DNA_repair,_bacterial | DNA repair exonuclease family protein YhaO | 0 | 0 | 0 | 1 | 1 | 2 | 1 | 1 | 1 | 3 | 1 | 2 |
|  |  | DNA_repair,_bacterial | Exodeoxyribonuclease VII large subunit (EC 3.1.11.6) | 13 | 15 | 18 | 2 | 25 | 16 | 8 | 13 | 7 | 0 | 5 | 13 |
|  |  | DNA_repair,_bacterial | DNA polymerase IV (EC 2.7.7.7) | 3 | 3 | 8 | 3 | 8 | 2 | 3 | 8 | 3 | 1 | 0 | 3 |
|  |  | DNA_repair,_bacterial | Endonuclease IV (EC 3.1.21.2) | 0 | 1 | 3 | 1 | 6 | 2 | 4 | 5 | 0 | 0 | 1 | 2 |
|  |  | DNA_repair,_bacterial | Endonuclease V (EC 3.1.21.7) | 1 | 5 | 2 | 0 | 2 | 1 | 0 | 4 | 1 | 3 | 0 | 2 |
|  |  | DNA_repair,_bacterial | Exodeoxyribonuclease III (EC 3.1.11.2) | 64 | 54 | 46 | 13 | 57 | 103 | 64 | 43 | 41 | 37 | 39 | 51 |
|  |  | DNA_repair,_bacterial | Methylated-DNA--protein-cysteine methyltransferase (EC 2.1.1.63) | 15 | 10 | 18 | 6 | 19 | 26 | 13 | 11 | 7 | 17 | 10 | 23 |
|  |  | DNA_repair,_bacterial | DNA recombination protein RmuC | 7 | 0 | 0 | 0 | 6 | 11 | 2 | 0 | 0 | 0 | 6 | 1 |
|  |  | DNA_repair,_bacterial | Very-short-patch mismatch repair endonuclease (G-T specific) | 0 | 1 | 0 | 0 | 2 | 0 | 0 | 0 | 0 | 0 | 0 | 1 |
|  |  | DNA_repair,_bacterial | A/G-specific adenine glycosylase (EC 3.2.2.-) | 10 | 10 | 7 | 3 | 18 | 31 | 4 | 21 | 3 | 2 | 2 | 7 |
|  |  | DNA_repair,_bacterial | G:T/U mismatch-specific uracil/thymine DNA-glycosylase | 1 | 0 | 1 | 2 | 0 | 3 | 2 | 2 | 4 | 0 | 1 | 2 |
|  |  | ATP-dependent_Nuclease | ATP-dependent nuclease, subunit A | 0 | 2 | 3 | 0 | 0 | 7 | 3 | 0 | 3 | 0 | 3 | 9 |
|  |  | ATP-dependent_Nuclease | ATP-dependent nuclease, subunit B | 1 | 0 | 0 | 0 | 2 | 3 | 2 | 3 | 3 | 0 | 0 | 0 |
|  |  | DNA_repair,_bacterial_RecFOR_pathway | ATP-dependent DNA helicase RecQ | 54 | 78 | 70 | 15 | 120 | 88 | 36 | 47 | 22 | 10 | 30 | 69 |
|  |  | DNA_Repair_Base_Excision | ATP-dependent DNA ligase (EC 6.5.1.1) | 23 | 27 | 19 | 13 | 26 | 26 | 25 | 16 | 7 | 12 | 10 | 26 |
|  |  | DNA_Repair_Base_Excision | ATP-dependent DNA ligase (EC 6.5.1.1) clustered with Ku protein, LigD | 1 | 1 | 1 | 0 | 2 | 0 | 1 | 1 | 0 | 0 | 1 | 1 |
|  |  | DNA_Repair_Base_Excision | DNA ligase (EC 6.5.1.2) | 7 | 6 | 3 | 0 | 8 | 5 | 6 | 4 | 0 | 2 | 1 | 9 |
|  |  | DNA_Repair_Base_Excision | DNA-3-methyladenine glycosylase (EC 3.2.2.20) | 4 | 6 | 6 | 1 | 19 | 13 | 10 | 8 | 3 | 3 | 6 | 7 |
|  |  | DNA_Repair_Base_Excision | DNA-3-methyladenine glycosylase II (EC 3.2.2.21) | 5 | 4 | 3 | 2 | 8 | 8 | 6 | 5 | 2 | 2 | 1 | 4 |
|  |  | DNA_Repair_Base_Excision | Endonuclease III (EC 4.2.99.18) | 19 | 22 | 10 | 3 | 17 | 17 | 10 | 10 | 6 | 2 | 2 | 16 |
|  |  | DNA_Repair_Base_Excision | Flap structure-specific endonuclease (EC 3.-.-.-) | 0 | 1 | 0 | 0 | 2 | 2 | 3 | 1 | 0 | 0 | 0 | 0 |
|  |  | DNA_Repair_Base_Excision | Ku domain protein | 0 | 0 | 1 | 0 | 0 | 0 | 0 | 1 | 0 | 0 | 0 | 3 |
|  |  | DNA_repair,_UvrABC_system | Excinuclease ABC subunit A | 161 | 140 | 164 | 34 | 215 | 220 | 129 | 119 | 91 | 45 | 72 | 157 |
|  |  | DNA_repair,_UvrABC_system | Excinuclease ABC subunit B | 92 | 88 | 80 | 11 | 105 | 107 | 78 | 75 | 55 | 19 | 56 | 92 |
|  |  | DNA_repair,_UvrABC_system | Excinuclease ABC subunit C | 50 | 37 | 61 | 6 | 57 | 70 | 30 | 39 | 24 | 4 | 19 | 43 |
|  |  | DNA_repair,_bacterial_UvrD_and_related_helicases | ATP-dependent DNA helicase UvrD/PcrA | 10 | 4 | 6 | 0 | 14 | 10 | 3 | 6 | 4 | 2 | 2 | 9 |
|  |  | DNA_repair,_bacterial_UvrD_and_related_helicases | DNA helicase IV | 0 | 0 | 0 | 0 | 0 | 1 | 0 | 0 | 0 | 0 | 0 | 0 |
|  |  | DNA_repair,_bacterial_UvrD_and_related_helicases | ATP-dependent DNA helicase pcrA (EC 3.6.1.-) | 7 | 5 | 10 | 1 | 5 | 8 | 3 | 6 | 2 | 3 | 0 | 5 |
|  |  | DNA_repair,_bacterial_UvrD_and_related_helicases | ATP-dependent DNA helicase UvrD/PcrA, clostridial paralog 2 | 0 | 1 | 0 | 0 | 0 | 0 | 0 | 0 | 0 | 0 | 0 | 0 |
|  |  | DNA_repair,_bacterial_DinG_and_relatives | DinG family ATP-dependent helicase YoaA | 0 | 0 | 0 | 0 | 0 | 0 | 0 | 0 | 0 | 0 | 0 | 1 |
|  |  | DNA_repair,_bacterial_DinG_and_relatives | DinG family ATP-dependent helicase CPE1197 | 0 | 0 | 0 | 0 | 0 | 0 | 0 | 0 | 1 | 0 | 0 | 0 |
|  |  | 2-phosphoglycolate_salvage |  | 0 | 0 | 0 | 0 | 0 | 3 | 0 | 0 | 0 | 0 | 0 | 0 |
