# Supplementary material for: What Is the Role of Archaea in Plants? New Insights from the Vegetation of Alpine Bogs
Source: mSphere. 2018 May 9;3(3):e00122-18. doi: 10.1128/mSphere.00122-18 (PMC5956146; doi:10.1128/mSphere.00122-18)
Supplement: TABLE S3 [file sph003182536st3.docx]

| **archaeal functional annotations of SEED database** | **absolute abundance** | **rel. abundance [%]** |
| --- | --- | --- |
| total archaeal functional hits | 285058 | 100.00 |
| carbohydrates | 61456 | 21.56 |
| central carbohydrate metabolism | 21597 | 7.58 |
| amino acids and derivatives | 57274 | 20.09 |
| fatty acids, lipids and isoprenoids | 6670 | 2.34 |
| cofactors, vitamins, prosthetic groups, pigments | 18359 | 6.44 |
| fermentation | 4559 | 1.60 |
| one-carbon metabolism | 6148 | 2.16 |
| CO_2_ fixation | 2069 | 0.73 |
| nitrogen fixation | 20 | 0.01 |
| nitrogen metabolism | 2776 | 0.97 |
| ammonia assimilation | 1958 | 0.69 |
| stress response | 5797 | 2.03 |
| oxidative stress response | 2497 | 0.88 |
| DNA repair | 6541 | 2.29 |
| osmotic stress | 701 | 0.25 |
| motility and chemotaxis | 3967 | 1.39 |
| flagellar motility | 665 | 0.23 |
| glycogen degradation | 950 | 0.33 |
| auxin biosynthesis | 1946 | 0.68 |
